# Exploring the casual association between coffee intake and bladder cancer risk using Mendelian Randomization

**DOI:** 10.3389/fgene.2022.992599

**Published:** 2022-09-30

**Authors:** Yuqing Deng, Tingting Wu, Gang Luo, Lin Chen

**Affiliations:** ^1^ Department of Urology, The Central Hospital of Wuhan, Tongji Medical College, Huazhong University of Science and Technology, Wuhan, China; ^2^ Department of Thyroid Breast Surgery, The Central Hospital of Wuhan, Tongji Medical College, Huazhong University of Science and Technology, Wuhan, China

**Keywords:** bladder cancer, coffee consumption, Mendelian randomization, risk, causal association

## Abstract

**Objective:** Several observational studies have suggested that coffee consumption is associated with a lower risk of bladder cancer. However, observational studies are susceptible to confounding factors and reverse causality. We used a two-sample Mendelian randomization (MR) method to assess the causal nature of this association.

**Methods:** At the genome-wide significance level (*p* < 5 × 10^−8^), 12 single nucleotide polymorphisms (SNPs) strongly associated with coffee consumption were used as instrumental variables (IVs). Summary-level data on genetic variation in bladder cancer were obtained from the United Kingdom biobank (420,838 samples) and FinnGen consortium (175,121 samples). Multiple MR methods were used. Heterogeneity and horizontal pleiotropy were detected using Cochran’s Q test and MR-Egger.

**Results:** Twelve SNPs were included in the primary analysis. After excluding 8 SNPs with potential secondary phenotypes, the remaining 4 SNPs were included in the sensitivity analysis. In all analyses, Cochran’s Q statistic indicated that there was no heterogeneity among SNPs, and the MR-Egger analysis did not reveal the existence of horizontal pleiotropy (*p* > 0.05). In the United Kingdom Biobank, the odds ratio (OR) for bladder cancer was 1.022 (95% confidence interval (CI), 0.679–1.537) for per 50% increase in coffee consumption. Consistent results were obtained in the FinnGen consortium (OR = 0.890, 95% CI, 0.467–1.697). Sensitivity analysis showed consistent results with primary analysis.

**Conclusion** This study does not support a causal association between habitual coffee consumption and bladder cancer risk.

## Introduction

Bladder cancer is the 10th most common cancer worldwide (Sung et al., 2021). Incidence rates are consistently higher in men than women, although sex differences varied greatly between countries (Antoni et al., 2017). Bladder cancer ranks 13th in terms of deaths ranks (Antoni, et al., 2017).

Coffee is one of the most common beverages in the world (Ludwig et al., 2014; O'Keefe et al., 2018). A traditional cup of coffee could contain up to 1,000 bioactive compounds, including a wide variety of aromatic compounds, antioxidants, and most importantly, caffeine (Cornelis, 2019). A recent umbrella review considered data from 201 meta-analysis of epidemiological studies of 67 unique health outcomes, and concluded that coffee likely has a beneficial role in reducing risk of cardiovascular diseases, type 2 diabetes, Parkinson’s disease and several cancers, but that high caffeine intake is likely harmful on pregnancy outcomes, such as pregnancy loss and low birth weight (Poole et al., 2017). Overall, coffee intake within the normal intake range (i.e., 3 to 4 cups per day) appears to be safe and is more likely to have health benefits than harms (Poole, et al., 2017). In addition, an umbrella review of the evidence from meta-analyses was performed in the same year (Grosso et al., 2017). In the selected 112 meta-analyses of observational studies, coffee was associated with a probable decreased risk of cardiovascular disease, type 2 diabetes, Parkinson’s disease and several cancers (Grosso, et al., 2017). However, in the selected 9 meta-analyses of randomized controlled trials, coffee was associated with a rise in serum lipids (Grosso, et al., 2017). A large number of researchers are interested in the relationship between coffee consumption and cancer risk, given the antioxidant and anti-inflammatory properties of the beneficial ingredients in coffee (Bakuradze et al., 2010; Castaldo et al., 2021). Several observational studies have investigated the association between coffee intake and bladder cancer risk. However, inconsistent results have been produced, including reverse association (Sugiyama et al., 2017), positive association (De Stefani et al., 2007; Loftfield et al., 2017) and null association (Demirel et al., 2008; Kobeissi et al., 2013). A meta-analysis of 13 case-control studies (5,911 cases and 16,172 controls) found a positive association between coffee intake and bladder cancer among never-smokers (Yu et al., 2019). In addition, a meta-analysis of 12 cohort studies (501,604 participants) showed a positive association between coffee consumption and bladder cancer risk among male smokers, but not among female smokers (Yu et al., 2020). A meta-analysis including 16 prospective cohort studies found no statistical association between coffee intake and bladder cancer risk (Dai et al., 2019).

Bladder is an excretory organ and fluid intake may play an important role in the development of bladder cancer (Bai et al., 2014). The results of existing observational epidemiological studies could be influenced by confounding and reverse causation, making causal inference hard. Mendelian Randomization (MR) can strengthen causal inference of expose-outcome association by using genetic variation as instrumental variables of exposure (Burgess et al., 2015). The genetic variants are unlikely to be associated with confounding factors related to exposure and outcome because they are randomly combined at conception. In addition, MR design also reduces reverse causality because allele randomization precedes disease development (Hartwig et al., 2016).

Therefore, in this study, we used a two-sample MR method to explore the causal relationship between coffee intake and bladder cancer risk, and conducted a sensitivity analysis to further verify the reliability of the results.

## Methods

### Selection of genetic instrument variables

This study used publicly available summary-level data from large genome-wide association studies (GWASs), which did not involve individual patients and required no ethical review or informed consent. Single nucleotide polymorphism (SNPs) significantly associated with coffee intake came from a meta-analysis of four GWASs (United Kingdom Biobank and three United States cohorts) (Zhong et al., 2019). The meta-analysis of GWASs included 375,833 individuals of European ancestry, and adjusted for body mass index, gender, age, total energy, and top 20 principal components. In the United Kingdom Biobank, a questionnaire was used to collect all participants’ coffee intake at baseline: “How many cups of coffee (including caffeinated and decaffeinated coffee) do you drink per day?” In the United States cohort, a semi-quantitative food frequency questionnaire was used to collect regular and decaffeinated coffee intake. The median coffee consumption ranged from 1.1 to 2.5 cups per day. We identified 15 SNPs at the genome-wide association significance level (*p* < 5 × 10^−8^) (Zhong, et al., 2019). Selected SNPs explained about 0.48% of the phenotypic difference in coffee intake. The effect sizes for the SNP-coffee associations were expressed per 1% of increase in coffee consumption in the meta-analysis of GWASs. The effect sizes for the SNP-coffee associations were scaled to a 50% increase (eg, an increase from 1 cup to 1.5 cups). We calculated the linkage disequilibrium (LD) (r^2^ > 0.01 and clump window <1,000 kb) between the 15 SNPs based on LD reference panel from 1,000 Genomes of European populations. In this MR study, 12 independent SNPS were used as genetic instrumental variables for coffee intake after excluding 3 SNPs in LD (rs117692895, rs12699844 and rs4719497 on chromosome 7) and included in the primary analysis (Table 1). Rs2472297 in CYP1A1/2 and rs4410790 in AHR showed strong association with coffee intake. Then, to fulfill the MR condition that IVs were not affected by any confounding factors, we searched PhenoScanner V2 web site (http://www.phenoscanner.medschl.cam.ac.uk/) to identify whether these SNPs were associated with potential secondary phenotype (Supplementary Table S1). Eight SNPs with potential secondary phenotypes at the genome-wide association significance level (*p* < 5 × 10^−8^) were excluded (Table 1). The remaining 4 SNPs were used as instrumental variables for coffee intake and included in the sensitivity analysis.

**TABLE 1:** Characteristics of the single-nucleotide polymorphisms associated with coffee consumption.

Exposure	Chr	SNP	Included in the primary MR analysis	Included in the sensitivity MR analysis
Coffee consumption	1	rs574367	Yes	No, Horizontal pleiotropy
Coffee consumption	2	rs10865548	Yes	No, Horizontal pleiotropy
Coffee consumption	2	rs1260326	Yes	No, Horizontal pleiotropy
Coffee consumption	7	rs1057868	Yes	No, Horizontal pleiotropy
Coffee consumption	7	rs117692895	No, Linkage disequilibrium	No, Linkage disequilibrium
Coffee consumption	7	rs12699844	No, Linkage disequilibrium	No, Linkage disequilibrium
Coffee consumption	7	rs34060476	Yes	No, Horizontal pleiotropy
Coffee consumption	7	rs4410790	Yes	No, Horizontal pleiotropy
Coffee consumption	7	rs4719497	No, Linkage disequilibrium	No, Linkage disequilibrium
Coffee consumption	7	rs73073176	Yes	Yes
Coffee consumption	11	rs597045	Yes	Yes
Coffee consumption	14	rs1956218	Yes	Yes
Coffee consumption	15	rs2472297	Yes	No, Horizontal pleiotropy
Coffee consumption	18	rs66723169	Yes	No, Horizontal pleiotropy
Coffee consumption	22	rs2330783	Yes	Yes

### Data source for bladder cancer

The summary-level genetic data on bladder cancer was obtained from the United Kingdom Biobank, which recruited about 500,000 adults aged 37–73  years at the baseline of recruitment between 2006 and 2010. In the United Kingdom Biobank, 420,838 men and women of European participants (2,883 bladder cases and 417,955 controls) with a median age of 63 years were included. Cases of bladder cancer were defined by the codes of the International Classification of Diseases (ICD-9) and ICD-10 (including trigone, dome, lateral wall, anterior wall, and posterior wall of bladder, bladder neck, ureteric orifice, urachus, and overlapping lesion of bladder). The GWAS was performed with the adjustment for age, sex, and the first ten genetic principal components by logistic regression. To verify the reliability of the results, summary-level genetic data on bladder cancer were also obtained from the fifth release of FinnGen consortium, which included up to 175,121 men and women of Finnish descents (1,115 bladder cancer cases and 174,006 controls with a median age of 61 years) after the removal of individuals with ambiguous gender, high genotype missingness (>5%), excess heterozygosity (±4 SD), and non-Finnish ancestry. All genetic association estimates were calculated by logistic regression with the adjustments of genetic principal components, and relevant covariates, such as age, sex and genotyping batch (Table 2).

**TABLE 2:** Details of studies and datasets used for analyses.

Exposure or outcome	Study or consortium	Participants	Ethnicity	Web source
Coffee consumption	Zhong VW	375 833	European	https://academic.oup.com/hmg/article/28/14/2449/5424254
Bladder cancer	FinnGen	1 115/174 006	European	https://storage.googleapis.com/finngen-public-data-r5/summary_stats/finngen_R5_C3_BLADDER_EXALLC.gz
Bladder cancer	United Kingdom Biobank	2 883/417 955	European	https://pan-ukb-us-east-1.s3.amazonaws.com/sumstats_flat_files/icd10-C67-both_sexes.tsv.bgz

### F statistics

To further verify the correlation hypothesis, F statistic was used to evaluate whether the selected instrumental variable have weak instrumental variable bias (Pierce et al., 2011). The calculation formula is $=R^{2}N-2/1-R^{2}$ , where, N is the sample size of exposure, and *R*2 is the proportion of exposure variation explained by instrumental variables.

### Mendelian randomization analysis

In this study, fixed and random effect inverse variance weighted (IVW), weighted median and MR-Egger regression methods were used for MR analysis (Burgess et al., 2013). If all SNPS selected are valid instrumental variables, IVW method can provide unbiased estimation (Burgess, et al., 2013). Assuming that no more than 50% of the estimated MR effect weights are derived from multi-effect SNPS, the weighted median method gives consistent effect estimates, where the weights are determined by the strength of their relationship to exposure (Bowden et al., 2016). The MR-Egger regression examines the horizontal pleiotropy of instrumental variables and provide estimates after correcting for pleiotropy (Bowden et al., 2015). If the intercept of the regression equation is 0 or the *p*-value of the intercept term is not significant, no horizontal pleiotropy is suggested. MR pleiotropy residual sum and outlier (MR-PRESSO) method was used to detect the presence of horizontal outliers. Cochran’s Q was used to verify the heterogeneity between the causal estimates of each SNPs in the IVW and MR-Egger methods. The leave-one-out method was used to exclude each SNP one by one and calculate the combined effect of remaining SNPs, so as to determine whether the causal association between exposure factors and outcome variables is caused by the main effect of an instrumental variable. R (Version 3.6.1) software with MR-PRESSO (Version 1.0) and Two-Sample MR (Version 0.5.5) packages were used for statistical analysis, and *p* < 0.05 was considered statistically significant.

## Results

### Weak instrumental variable bias, outlier detection, heterogeneity and horizontal pleiotropy test

**FIGURE 1:**
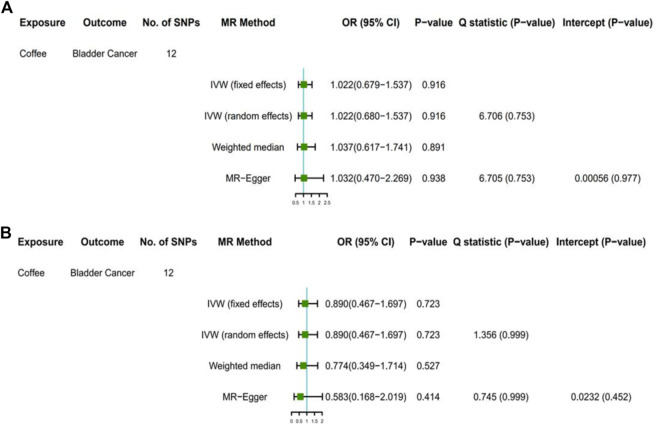
Results of primary analyses. Association of genetically predicted coffee consumption with bladder cancer risk in the United Kingdom Biobank **(A)** and FinnGen consortium **(B)** OR, odds ratio; CI, confidence interval; SNP, single-nucleotide polymorphism.

**FIGURE 2:**
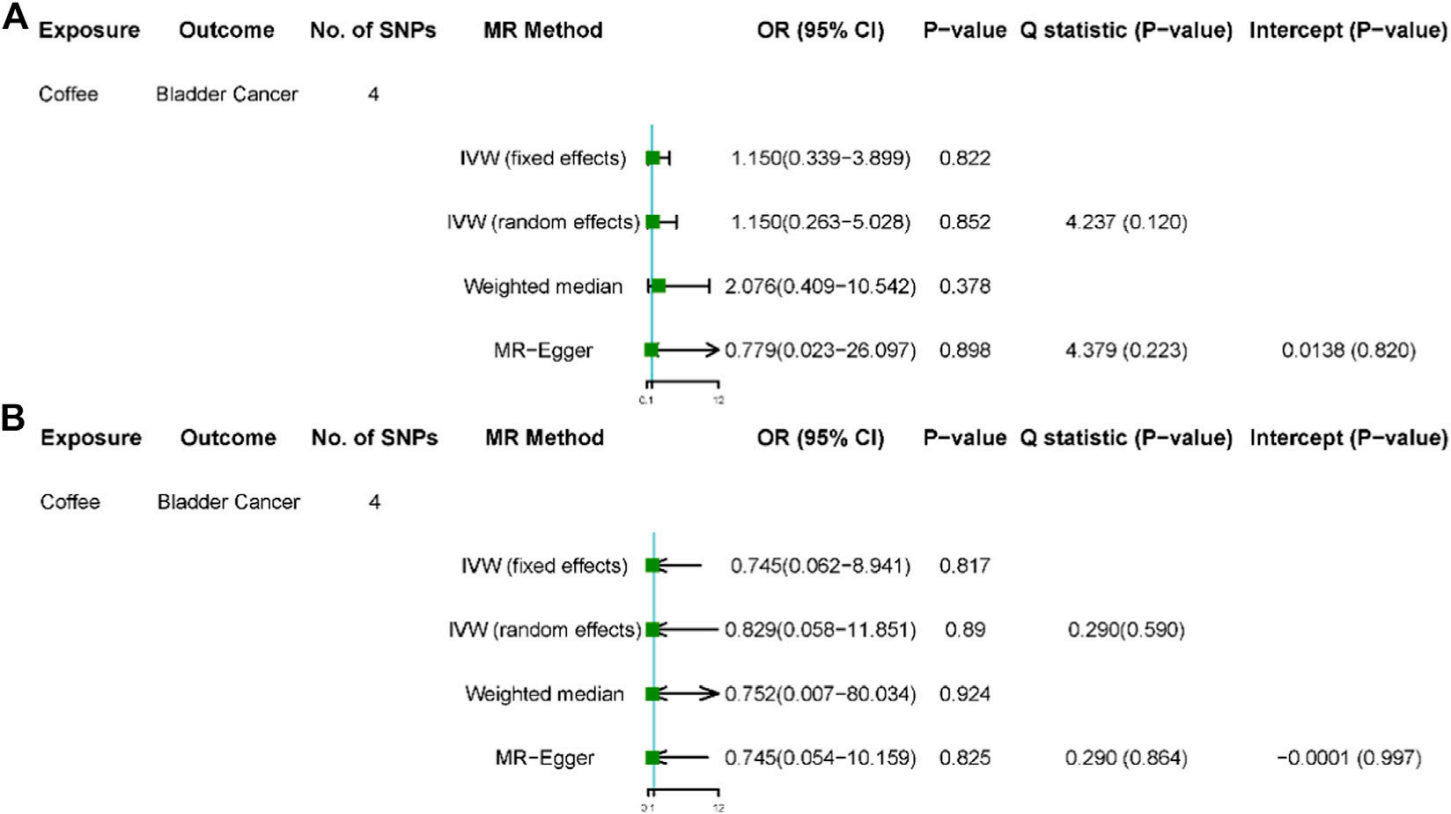
Results of sensitivity analyses. Association of genetically predicted coffee consumption with bladder cancer risk in the United Kingdom Biobank **(A)** and FinnGen consortium **(B)**. OR, odds ratio; CI, confidence interval; SNP, single-nucleotide polymorphism.

The F statistic of coffee intake was 159. When the F statistic was more than 10, the possibility of weak instrumental variable was relative small (Pierce, et al., 2011). Therefore, the results of MR analysis were unlikely to be affected by weak instrumental variables. No outliers were observed in the MR-PRESSO analysis and sensitivity analysis (*p* > 0.05). In the primary analysis and sensitivity analysis, Cochran’s Q statistics showed that there was no heterogeneity among SNPs (all *p* > 0.05), and no horizontal pleiotropy was found in the MR-Egger analysis (*p* > 0.05) (Figure 1 and Figure 2).

### Primary analysis of two-sample mendelian randomization

Among GWAS using the United Kingdom Biobank for bladder cancer, the odds ratio (OR) of bladder cancer risk was 1.022 (95% CI, 0.679–1.537) for each 50% increase in coffee intake by the fixed-effect IVW method (Table 3, Figure 1A). The results based on random-effect IVW, weighted Median and MR-Egger showed that, for each 50% increase in coffee intake, the ORs of bladder cancer risk were 1.022 (95% CI, 0.680, 1.537), 1.037 (95% CI, 0.617, 1.741) and 1.032 (95% CI, 0.470, 2.269), respectively. Consistent results were obtained in the FinnGen consortium (fixed effect IVW: OR = 0.890, 95%CI, 0.467–1.697; random effect IVW: OR = 0.890, 95% CI, 0.467–1.697; weighted median: OR = 0.774, 95% CI, 0.349–1.714; MR-Egger: OR = 0.583, 95% CI, 0.168–2.019) (Figure 1B).

**TABLE 3:** Association of coffee consumption and risk of bladder cancer.

SNP	EA	NEA	EAF	Coffee consumption			Bladder cancer (United Kingdom biobank)			Bladder cancer (FinnGen)		
				Beta	SE	*P*	Beta	SE	*P*	Beta	SE	*P*
rs1057868	T	C	0.29	1.97	0.16	5.26E-33	−0.017290	0.03113	0.5786	−0.0068	0.0444	0.8777
rs10865548	G	A	0.83	1.54	0.19	4.46E-15	0.82880	0.003879	0.03740	0.0233	0.0585	0.6898
rs1260326	C	T	0.61	1.36	0.15	2.62E-19	−0.010470	0.02884	0.7166	0.0172	0.0455	0.7054
rs1956218	G	A	0.56	0.82	0.15	3.62E-08	0.015560	0.02851	0.5853	−0.0096	0.0439	0.8267
rs2330783	G	T	0.99	4.53	0.63	1.57E-12	−0.164900	0.11960	0.1680	−0.1408	0.2665	0.5972
rs2472297	T	C	0.27	4.54	0.17	5.19E-155	−0.004760	0.03188	0.8813	−0.0232	0.0501	0.6429
rs34060476	G	A	0.13	1.89	0.22	5.06E-18	−0.022420	0.04151	0.5892	−0.0019	0.0636	0.9759
rs4410790	C	T	0.63	3.94	0.15	5.59E-141	0.63940	0.022060	0.02931	−0.0213	0.0461	0.6441
rs574367	T	G	0.21	1.05	0.18	8.06E-09	0.21660	0.027640	0.03450	0.0205	0.056	0.7139
rs597045	A	T	0.69	1.07	0.16	6.62E-11	−0.0210	0.0310	0.503	0.021	0.0461	0.649
rs66723169	A	C	0.23	1.47	0.18	9.88E-17	−0.018810	0.03359	0.5755	0.0118	0.0573	0.8365
rs73073176	C	T	0.87	2.31	0.22	5.56E-25	0.13640	0.056390	0.04224	0.0099	0.0762	0.8969

### Sensitivity analysis of two sample mendelian randomization

**FIGURE 3:**
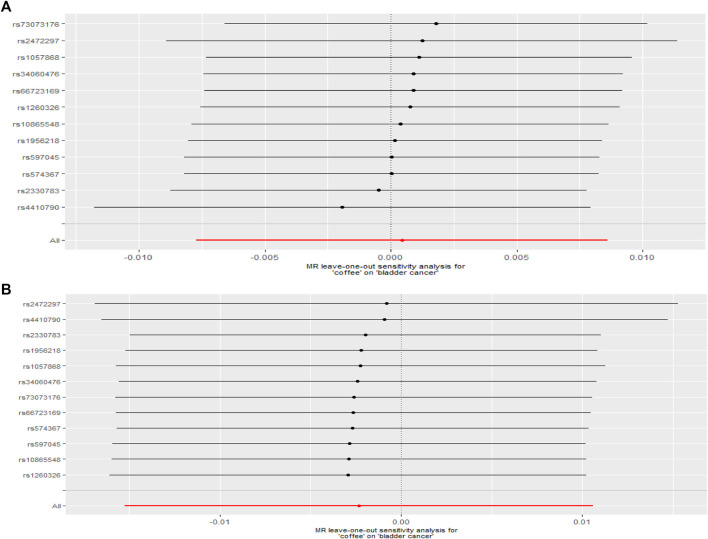
Leave-one-out analysis. **(A)** United Kingdom Biobank **(A)** and **(B)** FinnGen consortium.

After removed potential secondary phenotypes associated with genetic variations in coffee intake, sensitivity analysis showed consistent results with the primary analysis (Figures 2A,B). In addition, after 12 SNPs were excluded one by one, the combined effect values of the remaining 11 SNPs were similar with the primary analysis, indicating that the result was not significantly affected by a single SNP, that is, the choice of instrumental variables in our study is reasonable (Figure 3).

## Discussion

Our study evaluated the effect of coffee intake on the risk of bladder cancer using a two-sample MR analysis. No significant association was found between genetic predisposition to coffee consumption and bladder cancer risk using summary-level data of large scale GWAS. No horizontal pleiotropy or outlying SNP was found in this study. In addition, the results were robust in the sensitivity analyses.

Coffee is a compound of more than 1,000 biologically active ingredients, including caffeine and various minerals. Many of these compounds have been shown to have anti-tumor potential (Ludwig, et al., 2014). However, previous studies based on the association between coffee consumption and bladder cancer risk have yielded inconsistent results. In a dose-response meta-analysis, the relative risk (RR) for bladder cancer was 1.01 (95% CI: 0.98–1.03) for each 1 cup/day increase in coffee intake, and there was no evidence for a non-linear association between coffee intake and bladder cancer risk (Dai, et al., 2019). However, in a case-control study, coffee intake of 1 cup/day, 2 cups/day, 3 cups/day, and 4 cups/day was associated with an RR of 1.07(95% CI:1.02–1.13), 1.15 (95% CI: 1.05 1.26), 1.22 (95% CI: 1.08 1.38) and 1.29 (95% CI: 1.12 1.48) for bladder cancer in non-drinkers after adjusted for smoking status (Wijarnpreecha et al., 2017). However, those findings were majorly based on cross-sectional and case-control studies and therefore were prone to residual confounding from other coffee intake-correlated lifestyles (such as diet composition of protection) and behaviors as well as misclassification bias. In addition, observational studies that measure long-term coffee intake may be inaccurate.

A major strength of our study is the MR study design. Compared with randomized controlled trials and observational studies, MR design is not affected by confounding factors (such as social environment and lifestyle factors) and reverse causal association by using genetic variations as proxies. Genetic variation is long-term and stable. To our knowledge, this is the first two-sample MR study to assess the potential association between coffee consumption and bladder cancer risk. In this study, we excluded 3 SNPs in the LD analysis, and the MR-Egger test showed that the selected 12 SNPs had no horizontal pleiotropy, which increased the credibility of our study. In addition, in the sensitivity analysis, we excluded 4 SNPs that were not associated with other traits. The results of sensitivity analysis were consistent with primary analysis, suggesting that the association was not affected by confounders between coffee-related SNPs and bladder cancer.

One limitation is that the standard MR design assumes a linear relationship between exposure and outcome. Given that our MR study evaluated the relationship between coffee intake and bladder cancer in a linear assumption, the findings of our study could not rule out the possibility that moderate rather than excessive coffee intake may play a protective role on bladder cancer risk. The association could not be detected if there was a non-linear relationship or threshold effect between coffee consumption and bladder cancer. In addition, the study was limited to European population, and our conclusion may not be applicable to other populations, such as Asians. Another limitation of this study is that the association between different types of coffee and bladder cancer risk may differ due to the heterogeneity in the amount of caffeine and other components in different types of coffee. The hypothesis could not be verified in this MR study, because the genetically predicted coffee intake was associated with any type of coffee, including instant and filtered, as well as latte, espresso and other types of coffee.

In conclusion, we used SNPs as instrumental variables to determine the causal relationship between coffee intake and bladder cancer risk. There was no causal association between genetically predicted coffee intake and bladder cancer risk in the absence of confounding bias and horizontal pleiotropy. Several points need to be paid attention to in the future research. First, future MR studies need to assess the non-linear association between coffee intake and bladder cancer risk. Second, the relationship between the bioactive ingredients in coffee and bladder cancer is worth studying. Finally, the effect of different coffee types on bladder cancer risk needs to be studied.

## Data Availability

The original contributions presented in the study are included in the article/Supplementary Material, further inquiries can be directed to the corresponding authors.
