# Supplementary material for: Exploring the casual association between coffee intake and bladder cancer risk using Mendelian Randomization
Source: Front Genet. 2022 Sep 30;13:992599. doi: 10.3389/fgene.2022.992599 (PMC9565034; doi:10.3389/fgene.2022.992599)
Supplement: Supplementary file 1 [file Table1.docx]

Supplementary Material

# Supplementary Table

Supplementary Table 1. Potential secondary phenotypes of the genetic variants used for coffee consumption (*P*<5×10^-8^)

| SNP | Trait^1^ |
| --- | --- |
| rs2472297 | Creatinine in urine  Leg fat percentage left  Leg fat percentage right  Impedance of arm right  Potassium in urine  Platelet distribution width |
| rs66723169 | Body mass index  Alcohol intake frequency  Arm fat mass left  Arm fat mass right  Comparative body size at age 10  Comparative height size at age 10  Forced expiratory volume in 1-second, predicted  Height  Hip circumference  Impedance of arm left  Impedance of arm right  Impedance of leg left  Impedance of leg right  Impedance of whole body  Leg fat mass left  Leg fat mass right  Leg fat percentage left  Leg fat percentage right  Leg fat-free mass left  Leg fat-free mass right  Leg predicted mass left  Leg predicted mass right  Waist circumference  Whole body water mass  Worrier or anxious feelings  Coronary artery disease |
| rs574367 | Body mass index  Childhood body mass index  Body mass index in female non-smokers  Body mass index in non-smokers  Body mass index in smokers  Age at menarche |
| rs1260326 | Granulocyte count  Granulocyte percentage of myeloid white cells  High light scatter percentage of red cells  High light scatter reticulocyte count  log eGFR creatinine  Type II diabetes  Height  Total cholesterol  Triglycerides  2 hour glucose  Albumin  C reactive protein  FVII activity  FVII in plasma  Fasting blood glucose  Fasting insulin  Gamma glutamyl transferase  HDL cholesterol mean size lipoprotein fraction concentration  Hypertriglyceridemia  IFT172 expression in Lymphocytes lymphoblastoid cell lines tissue  IFT172 gene expression in adipose tissue  Serum creatinine estimated glomerular filtration rate eGFR  Serum urate  Triglycerides  Uric acid  Alcohol consumption  Cardiovascular disease risk factors |
| rs10865548 | Body mass index females  Body mass index males  Body mass index  Nonsyndromic striae distensae stretch marks  Age at menarche  Arm fat mass left  Arm fat mass right  Arm fat percentage left  Arm fat percentage right  Arm fat-free mass left  Arm fat-free mass right  Weight  Whole body fat mass  Whole body fat-free mass  Whole body water mass |
| rs4410790 | Habitual caffeine consumption  Habitual caffeine consumption caffeinated coffee intake  Habitual caffeine consumption female  Habitual caffeine consumption never smokers  Creatinine in urine  Potassium in urine  Sodium in urine |
| rs34060476 | Granulocyte percentage of myeloid white cells  High light scatter percentage of red cells  High light scatter reticulocyte count  Monocyte percentage of white cells  Reticulocyte count  Reticulocyte fraction of red cells  Triglycerides  Arm fat-free mass left  Arm fat-free mass right  Arm predicted mass left  Arm predicted mass right  Basal metabolic rate  Hip circumference  Impedance of arm left  Impedance of arm right  Impedance of leg right  Impedance of whole body  Leg fat-free mass left  Leg fat-free mass right  Leg predicted mass left  Leg predicted mass right  Self-reported gout  Sitting height  Sodium in urine |
| rs1057868 | Mean platelet volume  Creatinine in urine |

Abbreviation: SNP, single nucleotide polymorphism; *P*-value: *P*-value for the genetic association；

^1^ Similar traits were only listed once.
